# An Overview of Appropriate Medical Practice and Preparedness in Radiation Emergency Response

**DOI:** 10.7759/cureus.61627

**Published:** 2024-06-03

**Authors:** Akram Al-Ibraheem, Serin Moghrabi, Ahmed Abdlkadir, Heba Safi, Ziad Kazzi, Batool Al-Balooshi, Khaled Salman, Aysar Khalaf, Majdi Zein, Huda Al Naemi, Hanan Aldousari, Layth Mula-Hussain, Malik Juweid, Jun Hatazawa, Feras Hawwari, Asem Mansour

**Affiliations:** 1 Nuclear Medicine, King Hussein Cancer Center (KHCC), Amman, JOR; 2 Nuclear Medicine and PET/CT, King Hussein Cancer Center (KHCC), Amman, JOR; 3 Health and Environment Unit, World Health Organization, Amman, JOR; 4 Emergency Medicine, Emory University, Atlanta, USA; 5 Nuclear medicine, Dubai Health Authority, Dubai, ARE; 6 Department of Nuclear Medicine and PET/CT imaging, King Abdullah Medical City (KAMC), Makkah, SAU; 7 Department of Nuclear Medicine, Warith International Cancer Institute, Karbala, IRQ; 8 Department of Nuclear Medicine, Assad University Hospital, Damascus, SYR; 9 Nuclear Medicine, Hamad Medical Corporation, Doha, QAT; 10 Molecular Imaging Department, Jaber Alahmad Center for Molecular Imaging, Kuwait City, KWT; 11 Department of Radiation Oncology, Ninevah University, Mosul, IRQ; 12 Department of Radiation Oncology, Dalhousie University, Halifax, CAN; 13 Department of Radiology and Nuclear Medicine, Jordan University Hospital, Amman, JOR; 14 Department of Nuclear Medicine and Tracer Kinetics, Osaka University, Osaka, JPN; 15 Section of Pulmonary and Critical Care, Department of Internal Medicine, King Hussein Cancer Center (KHCC), Amman, JOR; 16 Radiology, King Hussein Cancer Center (KHCC), Amman, JOR

**Keywords:** public health, contamination, radiation practices, preparedness, radiation medicine, radiation emergency

## Abstract

Radiation emergencies involving high doses of nuclear radiation pose significant risks from exposure to ionizing radiation in various scenarios. These situations include transportation accidents involving radioactive materials, occupational exposure, nuclear detonations, dirty bombs, and nuclear power plant accidents. In addition to the immediate risks of acute radiation syndrome (ARS) and related diseases, long-term exposure can increase the risk of other health issues such as cardiovascular disease and cancer. Vulnerable populations, including pregnant women and children, face particular concern due to potential impacts on their health and the health of unborn babies.

The severity of ARS depends on several factors such as radiation dose, quality, dose rate, exposure uniformity, and individual biological responses. Bioindicators are biological responses or markers that help assess the severity and effects of radiation exposure on an individual. Bioindicators can include physical symptoms such as nausea, vomiting, and diarrhea, or laboratory tests such as changes in blood cell counts and gene expression that can help in assessing and treating exposed individuals. Additionally, early prodromal symptoms such as vomiting, diarrhea, and erythema can provide important clues for diagnosis and treatment. Developing a comprehensive plan for radiation emergencies is vital for safeguarding public health, infrastructure, and the environment.

First responders play a critical role in establishing safety perimeters, triage, and coordination with various stakeholders. Education and training are essential for medical personnel and the public. This article provides general recommendations and identifies challenges to effective radiation emergency preparedness and response.

## Introduction and background

While nuclear radiation emergencies can pose significant hazards to human life, health, property, and the environment, the majority involve exposure to low-to-medium levels of ionizing radiation. High-dose emergencies, such as those experienced by cleanup workers in the immediate aftermath of large-scale accidents like Chernobyl, are rare. Emergencies may arise from various scenarios including transportation accidents involving radioactive materials, occupational exposure in healthcare or research settings, and rare events such as nuclear detonations, dirty bomb incidents, or nuclear power plant accidents [1]. During such events, the population may experience radiation damage, resulting from the incorporation, external contamination, and/or external irradiation of various parts of the human body [2,3].

The initial damage of acute radiation emergency is caused by exposure to high levels of radiation, which can lead to acute radiation syndrome (ARS) and its related diseases, including the hematological, gastrointestinal, dermatological, or neurological syndrome. The severity of ARS is related to the dose of radiation exposure, but other factors, such as radiation quality, dose rate, homogeneity of exposure, and biological processes, also affect the outcome. For instance, cells and tissues respond differently to the same radiation dose, and several important biological processes have been identified with a strong impact on cell survival or cell death [4,5]. The consequences of the quality of treatment depend on the severity of ARS and the availability of medical resources and expertise. The use of bioindicators of effect, such as changes in blood cell counts, gene expression analysis, or proteomic and hematology biomarkers, might enable the integration of exposure and biological characteristics and provide improved clinical outcomes in exposed individuals [6,7]. Multiple organ dysfunction syndrome (MODS) can be a potential consequence of ARS, which is a critical condition that can occur during a nuclear emergency, where two or more organ systems fail to support the body's needs. It is a serious medical emergency that can be fatal without immediate treatment, including life support. MODS can affect any organ in the body, but the most commonly affected organs include the lungs, heart, kidneys, liver, brain, and blood [7].

Developing a comprehensive plan for an acute radiation emergency is of paramount importance for numerous compelling reasons. First and foremost, such a plan serves as a proactive safeguard for the safety and well-being of the public, medical personnel, and emergency responders. By detailing the steps to be taken in the event of a radiation emergency, it ensures a coordinated and efficient response. In addition, a well-structured plan includes the necessary protocols for radiation assessment, response strategies tailored to different types of radiation and radionuclides, and procurement of essential resources such as personal protective equipment (PPE), like, lead aprons, thyroid shields, protective gloves, and leaded glasses to shield themselves from harmful radiation exposure. Additionally, respiratory protection in the form of N95 masks or powered air-purifying respirators (PAPRs) is essential to prevent inhalation of radioactive particles. Furthermore, full-body suits made of materials impermeable fabrics are employed to minimize skin contact with radioactive contaminants. Dosimeters are crucial tools used to monitor radiation exposure levels, ensuring that health workers do not surpass safe limits [8]. These elements are critical to minimizing the immediate and long-term health risks associated with radiation exposure. Ultimately, a well-developed plan protects not only human lives, but also critical infrastructure, the environment, and the overall resilience of communities in the face of this complex and high-risk threat.

The first responders play a critical role in handling emergencies at the scene of an accident. First, it is essential to establish safety and security perimeters around the accident site. Next, triage becomes a crucial process, but its application may vary depending on the specific scenario and the number of victims involved. Triage should prioritize assessing and addressing immediate life-threatening injuries alongside classifying victims based on their level of radiation exposure, recognizing the importance of a balanced approach in managing diverse aspects of the emergency. The first responders must have adequate knowledge to assess and stabilize injuries among the different categories. Moreover, the emergency response plan should take into account coordination between various stakeholder groups and best-practice tools for emergency medical response, including referral hospitals, clinical and bio-dosimetry laboratories, and public health resources.

Radiation emergency preparedness encompasses the crucial aspect of providing thorough education and training to medical personnel and the public in the protocols for managing radiation emergencies. While it’s essential to emphasize the importance of training, it’s worth noting that certain radiation emergencies, particularly those involving environmental contamination or contamination of individuals, may involve complex factors beyond the scope of training alone, as evidenced in the Chernobyl accident in 1986 [9]. There is a need to develop credible and authentic guidance for the public about radiation-related incidents, actions to take in emergencies, and self-protection from short- and long-term dangers of radiation exposure.

This article aims to discuss the general guidelines and measures required to prepare for radiation emergencies and ensure expeditious management of the situation. Furthermore, various identified obstacles are examined and assessed to ensure optimal protection of the population and the environment.

## Review

Understanding ionizing radiation

**Figure 1:**
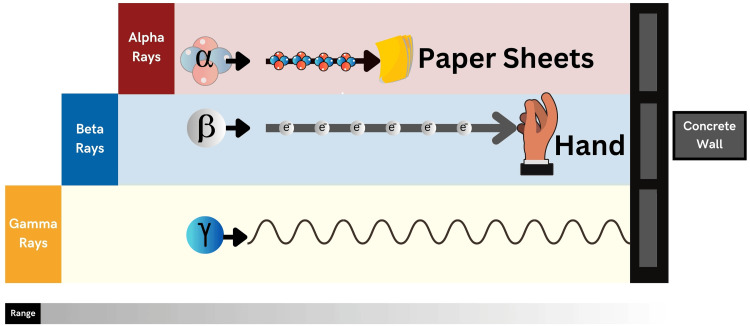
The Penetration Range of Alpha, Beta, and Gamma Radiation This figure is the original work of the authors.

Ionizing radiation is generated by either particles or electromagnetic waves that possess sufficient energy to disengage electrons from atoms or molecules, thus resulting in their ionization [10]. The extent and type of ionization are contingent upon the energy levels of the particles, rather than their quantity. Alpha and beta particles, neutrons, and cosmic rays are among the types of ionizing particles. Knowing the type of radiation is important in radiation emergencies because it helps estimate the damage, measure the dose, and estimate the affected service area. An alpha particle, for instance, is so heavy and energetic that it loses its energy over short distances in the air, cannot be measured by a gamma meter, and poses a significant health risk if incorporated (Figure 1).

As with alpha particles, beta particles may be blocked by clothing but can penetrate the skin and cause burns. The greatest potential risk associated with beta particles arises when they are inhaled, ingested, or enter the body through injection, as these routes of incorporation expose individuals to their harmful effects. Gamma rays travel tens of meters through the air can easily penetrate the human body and damage internal organs, and can be assessed by in situ spectrometry. Neutrons are extremely penetrating, making them a hazard during the initial blast and in the aftermath of accidents like Tokaimura, where they can continue to pose a risk to human health for an extended period [11,12]. Types of radionuclides produced in nuclear reactors, which pose major health and environmental effects, are either alpha emitter (e.g., plutonium-239, americium-241) or beta-gamma emitter (e.g., cesium-137, cobalt-60, and iodine-131) [13].

Cesium-134, cesium-137, and strontium-90 are the main sources of contamination in nuclear events, as they emit gamma rays that cause tissue damage by destroying cell molecules [14]. Moreover, they remain in the environment for a long time, as their physical half-lives are 2.1, 30, and 28.8 years, respectively [15,16]. While iodine-131 emits gamma rays and beta particles and has a short physical half-life of about 8 days, it can accumulate in the thyroid gland causing injury and damage [17,18].

The adverse health consequences of radiation exposure

The impact of radiation damage varies widely, ranging from minor and non-significant effects to life-threatening consequences, including both acute and long-term health implications [19]. Exposure to high levels of radiation can cause both acute and long-term health effects. Acute effects include skin burns and ARS, while long-term effects can include cancer and other somatic effects. While there is no conclusive evidence of radiation-induced genetic mutations in future generations, radiation damage can cause mutations in the DNA of exposed cells, which can lead to cancer and other health problems. On the other hand, radiation exposure found in the environment does not result in immediate health effects but does contribute significantly to the overall risk of cancer [20,21].

Biological Effects of Radiation Exposure

Nuclear radiation can cause harm to the tissues by causing deoxyribonucleic acid (DNA) damage, in both direct and indirect ways [19]. The damage is divided into two general types: tissue effects and stochastic effects. The tissue effects, such as skin erythema, have a minimum threshold radiation dose and require a specific number of cells to be affected before the changes become noticeable. With an increase in radiation, the likelihood and severity of the effects also increase. If the radiation dose is high enough, the effect is certain to occur [22,23]. Radiation-related cataracts are a type of cataract that can develop due to exposure to ionizing radiation, that falls in between the two categories. In contrast, stochastic effects like radiation-induced neoplasms do not have a detectable threshold dose [23]. The linear no-threshold (LNT) theory is a radiation risk model that suggests there is no safe threshold for exposure to ionizing radiation. It proposes that even the smallest doses of radiation carry some level of risk, particularly in terms of increasing the likelihood of cancer and other radiation-related health effects [23]. The theory asserts a linear relationship between radiation dose and risk, implying that the risk rises proportionally with the dose, without a dose threshold below which no harm is expected. While the LNT theory underpins radiation protection standards in many countries, it remains a subject of ongoing scientific debate and research, especially regarding very low radiation doses [22-25].

Clinical Effects of Radiation Exposure

Radiation accident can cause ARS, when the whole-body or significant partial-body irradiation exceeds 1 gray (Gy), which is an acute illness caused by irradiation of an external high dose of ionizing radiation in a short period to the entire body, causing depletion of immature parenchymal stem cells in specific tissues [26,27]. ARS is subdivided into four phases, depending on the time scale of symptoms (Table 1). During the manifest illness stage, the presence of each type of ARS is ultimately dependent on the absorbed radiation dose (Table 1).

**Table 1:** The Stages of Acute Radiation Syndrome (ARS) Adapted from reference [6].

ARS Stage	Onset	Duration	Presentation
Prodromal	1-72 hours	Several minutes- Several days	Non-specific but gastrointestinal symptoms predominate
Latent Stage	After prodromal stage	Few hours-few days	Temporary recovery from all signs and symptoms
Manifest Illness	After latent stage	Several days-several months	Include at least one of the following symptoms: Hematologic Syndrome: predominates when the absorbed radiation dose is between 2 and 10 gray. Gastrointestinal Syndrome: the full syndrome will usually occur with a dose > 10 gray Cardiovascular/Central Nervous System Syndrome: the full syndrome will usually occur with a dose > 50 gray. Death from this syndrome is usually the case especially with higher radiation doses.
Outcome	After manifest illness stage		Include one of the following outcomes: Full recovery: for those who will survive the manifest illness stage Death: for those who won’t survive during the manifest illness stage

While, local radiation injuries (LRI) can cause sub-syndrome of ARS, leading to cutaneous radiation syndrome (CRS), which typically arises from exposure to ionizing radiation that penetrates deeply into tissues or very large areas of skin from high-energy beta radiation. It can cause significant skin effects without necessarily affecting other subsyndromes of ARS (hematopoietic, gastrointestinal, neurovascular). LRI, depending on the body area exposed, may lead to various injuries and can result in partial exposure of highly radiosensitive vital organs [28].

Types of radiation exposure

External exposure occurs when individuals are near a radiation accident and are immediately exposed to high-energy photons such as gamma and X-rays, neutrons, and other particles [29]. It is noteworthy that direct exposure does not make the body radioactive, and therefore poses no threat to others. Although neutrons from a nuclear weapon detonation can cause slight radioactivity [30], it’s important to note that this induced radioactivity generally doesn’t present a direct health threat to healthcare providers.

Localized Exposure

Localized and profound exposure to radiation can occur due to the direct handling of sources with high radioactivity, which leads to LRI [31]. This type of exposure can cause cutaneous injury similar to burns, including blistering, erythema, desquamation, and ulceration, which often present about 12-20 days after irradiation with the onset and severity related to the magnitude of exposure. A local exposure of 3 Gy leads to second phase erythema, which refers to the main erythematous phase that clinically corresponds to a more severe reddening of the skin, and temporary epilation within 1-2 weeks, while a local exposure of 7 Gy may cause immediate effects like definitive epilation [28,31]. Vascular insufficiency may manifest after a significant period of time, leading to ulceration or tissue death in previously healed. Addressing localized radiation injuries typically involves measures to prevent infection, pain management, and vasodilation, and may require plastic surgery, grafting, or amputation in severe cases [32].

Total Body Exposure

Total body irradiation (TBI) refers to the exposure of the entire body to high levels of radiation, which can cause harm to cells throughout the body, particularly those that rapidly divide. TBI can occur due to exposure to low doses of radiation as well as high doses. It is widely acknowledged that certain tissues and organs within the body, including red bone marrow, gastrointestinal cells, and gonads, exhibit greater sensitivity or vulnerability to radiation-induced harm [33]. Symptoms of TBI can vary greatly and typically result in ARS.

Radiation contamination

Contamination refers to the unintentional deposition of radioactive substances in an area or surface where it is not intended [34]. It encompasses a broad spectrum of situations, from accidental releases in nuclear facilities to the aftermath of radiological incidents and emergencies. Radiation contamination can manifest in various forms, including the deposition of radioactive materials on surfaces, the incorporation of radionuclides into the human body, or environmental contamination in the form of soil, water, or air pollution. The presence of high levels of contamination can pose a danger to health, but medical professionals who adhere to the principles of radiation protection, use suitable protective gear, and maintain hygiene practices can reduce the risk of significant exposure or potential contamination.

Types of contamination

In the event of a radiation emergency, contamination may manifest as either external or internal.

External Contamination

This phenomenon occurs when radioactive contamination is deposited on the surface of the body or external objects, such as clothing. Effective management of such cases involves the prompt removal and control of the spread of radioactive material [34]. As such, it should be prioritized as a crucial component of early-stage radiation emergency response protocols.

Internal Contamination

Internal contamination can arise from the dispersion of radioactive material in the form of powders, liquids, or gases [34]. The entry of such material into the body can occur through inhalation, ingestion, skin penetration, or wounds and burns. The effectiveness of treatment is contingent on knowledge of the specific radionuclide and its chemical composition. Without prompt treatment, the efficacy of remediation may be constrained. Several approaches to the treatment of internal contamination exist, including reduction of absorption, dilution, blockage, displacement by non-radioactive substances, mobilization to facilitate elimination from tissues, and chelation.

Emergency response planning

Radiation emergencies warrant immediate actions by all relevant stakeholders, including governmental, non-governmental, private sectors, and international organizations. The emergency response plan outlines the organization and structure of the health sector’s response to emergencies [35]. A national radiation emergency response plan helps national counterparts to take systematic risk-informed actions, engage all key stakeholders, including communities, and establish a defined leadership to respond to emergencies and disasters at all levels [35]. This is one of the key attributes for a country to be prepared for such emergencies as required under the International Health Regulations (IHR) 2005 [36].

The emergency response plan should cover all phases of an emergency response, including activation, grading, operations, and de-escalation. Consistent evaluation, examination, and revision of the plan are crucial to ensure its optimal functionality.

Dealing with radiation exposure

At Scene Response

A witness plays a crucial role in reporting a radiation accident and aiding in a prompt response. He can quickly notify emergency services, determine the location and severity of the incident, and provide crucial details about the number of people affected. Once a witness reports an accident, it is important to initiate a response to the radiation emergency as quickly as possible.

Initiating Radiation Emergency Response

The first responder, who is the first person to arrive at the scene of a radiation incident and has an official role in the accident response, is responsible for handling all aspects of the emergency at the scene, under the supervision of the on-scene controller. Providing mitigation measures, confinement, crowd management, coordination of all response units at the scene, initial recovery and cleanup operations, protection of emergency workers, and protective measures. With the help of the radiological assessor, the team is responsible for carrying out source recovery, cleanup, and decontamination operations, as well as, estimating and recording the dose received by emergency workers and/or the public [37].

As part of the radiological assessment process, the team should ensure the safety of people in the accident area by determining the approximate distance from the source that is safe. A safety perimeter should be established where the radiation dose rate is 100 microsieverts per hour (µSv/h). Additionally, a security perimeter should be set up beyond this safety boundary to prevent public interference with emergency response operations [37].

At Scene Triage

Triage in disaster response offers several significant advantages. First, it separates individuals in need of immediate life-saving care from those with less severe injuries, ensuring that critical cases are addressed promptly. Second, it helps alleviate the strain on medical facilities by identifying and prioritizing minor injuries, with a relatively small percentage of casualties requiring overnight hospitalization. Lastly, triage facilitates the fair and rational distribution of casualties across available hospitals, preventing overwhelming burdens on any single facility, often reducing the strain to a non-disaster level [38]. During the initial response phase, first responders can use the Simple Triage and Rapid Treatment (START) protocol for primary triage. The START protocol is a simple technique used by first rescuers arriving on the scene to quickly identify patients in need of immediate treatment and transportation. Triage takes priority over emergency treatment, and all victims will need to be tagged. Emergency care administered by START teams is restricted to opening airways, controlling severe hemorrhage, and elevating patient’s feet. Casualties will be tagged according to the seriousness of their conditions and placed into one of the following categories: Immediate (critical) = red tag, Delayed (urgent) = yellow tag, Minor (ambulatory) = green tag, and Deceased (expired) = black tag. During primary triage, responders should focus on speed in sorting patients and implementing their moving to the correct treatment locations. For categorizing patients at multiple casualty incidents, first responders use colored triage tape and/or tags. Tagging patients early helps in tracking them and their condition. All tags should be waterproof and color-coded with the triage categories clearly shown. Secondary triage (re-triage) is performed when patients enter a staging area [38,39].

Identification of radioactive source

The identification of radioactive sources in the context of an acute radiation emergency is a critical and multifaceted process central to effective response and public safety. This endeavor involves the rapid and accurate determination of the presence, type, and quantity of radioactive materials involved in the incident. First and foremost, it necessitates the deployment of specialized radiation detection equipment, including dosimeters, spectroscopy tools, and radiation survey meters, which enable responders to assess radiation levels and identify the nature of the radioactive source. Furthermore, the utilization of nuclear forensics techniques may provide valuable insights into the origin and characteristics of the materials involved. It is imperative to establish communication and coordination between various stakeholders, such as emergency services, radiation safety experts, and law enforcement, to ensure a systematic approach to radioactive source identification. Swift and precise identification serves as the foundation for informed decision-making, enabling the implementation of appropriate protective measures, evacuation plans, and medical responses, while mitigating the potential health risks and consequences associated with acute radiation emergencies [40].

Decontamination

Decontamination procedures typically commence by removing all clothing, which is then placed in a plastic bag for disposal. The primary focus is on assessing open wounds, as they serve as a direct pathway for potential internal radiation exposure. Wounds are thoroughly irrigated with saline, and any foreign bodies are carefully removed by healthcare professionals. Subsequently, the patient is promptly transferred to a hospital for a thorough evaluation to determine the necessity of more extensive scrubbing and debridement to ensure biological decontamination. Following this, attention is directed to surveying the face, facial orifices, and intact skin. In cases of facial contamination, swabs are taken from the nose and mouth to assess potential pulmonary and gastrointestinal contamination. Affected individuals are advised to gently blow their noses and wash their faces and hands using soap and water [34,41]. Each decontamination attempt should be properly documented [42].

Suspected individuals of internal contamination with no open wounds, should be referred as soon as possible for outpatient nonemergency management that includes a collection of body fluids and whole-body gamma-ray counting [34].

Evacuation and transportation

In the event of a radiation incident, it is crucial to promptly and safely evacuate individuals who have been exposed to radiation. This requires a team of professionals, including a physician, a nurse, a radiation safety officer, and ambulance personnel, to manage the evacuation process from the site of the accident to the hospital [43]. During transport, individuals' wounds and fractures should be stabilized and covered to prevent further injury and contamination. The main goal of the evacuation team is to transfer individuals to the hospital for medical attention, prioritizing those with severe injuries. Before the evacuation process begins, all necessary measures should be taken at the accident site, and the transport vehicles should be equipped with decontamination equipment to handle any contamination. PPE should also be worn by the evacuation team to prevent radiation exposure.

In-hospital management

During a radiological emergency, the primary focus should be on the medical stabilization and treatment of patients rather than decontamination efforts [44]. The risk of radiation exposure and contamination for staff is relatively low and can be mitigated through the application of standard practices and procedures that should be followed to prevent radiation exposure. Unlike chemical or biological agents, radioactive contamination is readily detectable when survey meters are adequately available and properly utilized.

Initiating Hospital Response

Hospitals commonly appoint an emergency response coordinator to facilitate an efficient and prompt response to emergencies. This individual is responsible for acknowledging the ambulance’s arrival and organizing the hospital’s reaction to the situation. The emergency response coordinator collaborates closely with both the emergency response and radiation response teams to guarantee that all essential precautions are taken to safeguard the patient's and healthcare personnel’s health and well-being.

In-Hospital Triage

**Figure 2:**
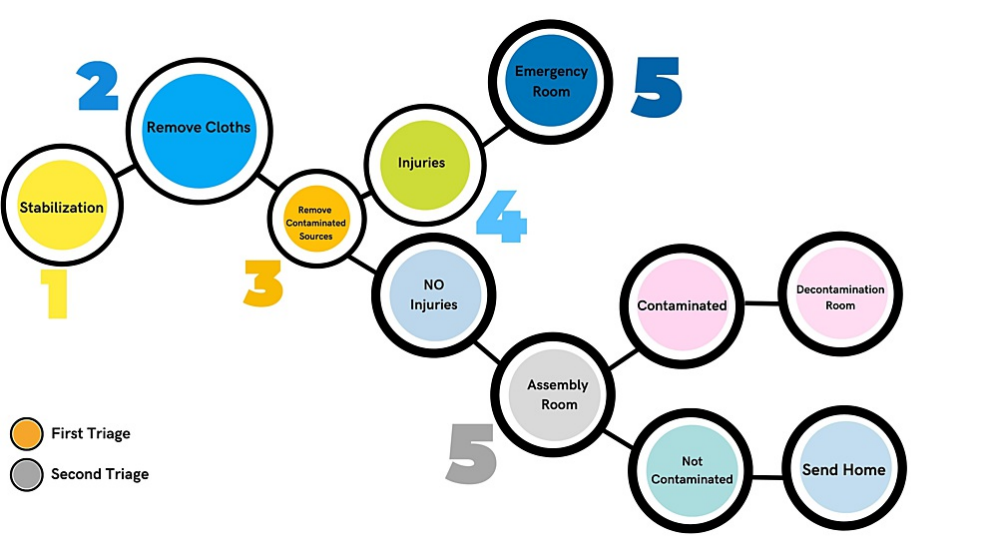
In-hospital Triage Diagram for Individuals with Radiation Accidents This figure is the original work of the authors.

Upon arriving at a hospital, individuals who have been exposed to radiation undergo two triage assessments to evaluate their condition (Figure 2).

The first triage is conducted immediately and comprises a thorough evaluation of the patient’s general state and vital signs. This triage is carried out by the hospital’s emergency response team, which comprises trained medical professionals such as doctors and nurses who specialize in addressing radiation emergencies. At this stage, individuals with critical injuries should be kept in the emergency room until further improvement is achieved. Otherwise, all other uninjured individuals will undergo a second in-hospital triage. Second, an in-hospital triage process is carried out by an expert team specialized in radiation response, comprising professionals such as trained radiation safety officers, dosimetrists, and radiation health physicists. The team is accountable for conducting a meticulous evaluation of the affected individual's radiation exposure, categorizing the type of radiation and the point of contamination entry, and selecting suitable decontamination and medical procedures.

Management at Radiation Emergency Room

Management at the emergency room following an acute radiation emergency is a multifaceted process that demands a systematic and coordinated approach. The initial steps involve the identification and triage of patients based on their radiation exposure levels, clinical symptoms, and the potential severity of their condition. Immediate medical care is critical for those with life-threatening symptoms of ARS, with a focus on addressing radiation-induced injuries and preventing or mitigating complications. Depending on the scale of the emergency, specialized teams, equipment, and facilities may be required to handle contaminated patients and those at risk of developing radiation-related complications [45].

For individuals brought to the hospital without undergoing decontamination at the scene, procedures must begin immediately to remove radioactive material from them to reduce the risk of further exposure to others and minimize harm to the patients themselves. This process includes thorough cleaning and careful documentation to confirm the successful removal of contaminants. Additionally, medical personnel must closely observe patients for radiation injuries, including organ-specific issues and the risk of secondary infections. The presence of contaminated patients in the hospital poses the risk of contaminating the hospital environment, which underscores the urgency of the decontamination process at the scene.

Assessment of clinical status and biochemical markers rather than relying solely on dose estimates plays a pivotal role in treatment planning and predicting the clinical course for affected individuals. This comprehensive assessment informs medical decisions, including the administration of medical countermeasures and the provision of supportive care. Acute radiation exposure can lead to a range of conditions, from hematological and gastrointestinal syndromes to skin and neurological effects. Timely and accurate diagnosis is essential to tailor treatments and interventions to individual patient needs.

Overall, the management at the emergency room after an acute radiation emergency requires a well-coordinated and interdisciplinary approach. Healthcare providers must be prepared to address not only the immediate medical needs of patients but also the long-term health effects and psychological impact of radiation exposure, emphasizing patient-centered care, safety, and the efficient use of available resources.

Management at Assembly Room

Once patients have been stabilized in the emergency room, they are subsequently transferred to the assembly room for further management. In this area, a comprehensive radiation survey is carried out using specialized detection equipment, such as Geiger Müller (GM) detectors and scintillation counters, to determine whether or not patients have been exposed to any external radioactive contamination. The radiation safety officer assumes responsibility for the assembly room and oversees the entire radiation survey process, while the radiological technologist assists with the survey. Medical personnel conduct clinical assessments of the patients.

Various diagnostics are conducted during this phase, such as urinalysis, eye and ear swabs, blood analyses, X-rays for stabilized fractures, and bronchoalveolar lavage if needed. The transfer of patients necessitating additional decontamination or those with internal contamination is determined by the outcomes of the radiation survey and biological dosimetry, and they are directed to the contamination room.

The assessment of radiation exposure typically involves several methods within the field of biological dosimetry, including cytogenetic analysis and the use of molecular biological markers. The cytogenetic approach involves a detailed examination of an individual's chromosomes to identify radiogenic changes resulting from radiation exposure. Meanwhile, molecular biological markers such as proteomics, transcriptomics, and the gH2AX assay can also provide insights into the biological effects of radiation exposure. These methods help to assess the severity and potential health outcomes of radiation exposure [46].

In addition to biological dosimetry, physical dosimetry (e.g., using radiation detection equipment) and clinical dosimetry (e.g., evaluating symptoms and health status) are important components in assessing radiation exposure (Table 2).

**Table 2:** Summary table for Medical Treatment Protocols for Radiation Accident Victims (METREPOL) Score System Adapted from reference [40].

	Score 1	Score 2	Score 3
Onset	Less than 12 hours	Less than 5 hours	Less than 30 minutes
Skin erythema	Nil	Present or absent	Significant
Asthenia	Mild	Moderate	Severe
Nausea	Mild	Moderate	Severe
Vomiting per day	Single episode	1-10 episodes	More than 10 episodes
Diarrhea per day	2-3 episodes; bulky	2-9; soft	More than 10; watery
Abdominal pain	Minimal	Intense	Excruciating
Headache	Nil	Present	Excruciating
Temperature	Normal	Low-grade fever	High-grade fever
Consciousness	Intact	Intact	Comatose
Blood pressure	Normal	Normal or slightly below normal	Significant hypotension
Actual Lymphocyte Count (ALC)			
ALC At 24 hours	Above 1500/mcl	Below 1500/mcl	Below 500/mcl
ALC At 48 hours	Above 1500/mcl	Below 1500/mcl	Below 100/mcl
Management Plan			
Provide care at	Outpatient settings	Inpatient	Inpatient

Together, these approaches provide a comprehensive understanding of an individual’s exposure and guide medical management and treatment planning [47].

Management at Decontamination Room

As stated before, the identification of internal contamination is accomplished utilizing bioassay. This involves withdrawing biological samples from suspected areas of internal contamination. Decontamination diagnostics may include nasal swabs from both nostrils, urine, blood, and fecal sampling.

Upon entering the body, radioisotopes behave similarly to stable isotopes of the same element from a chemical perspective. Hence, managing internal contamination necessitates a comparable approach to treating poisoning. Internal decontamination ultimately requires a multidisciplinary effort and is best executed by the collaborative work of emergency physicians, nurses, physicists, and medical toxicologists. Standard detoxification and decontamination methods, including antacids and cathartics (for instance, castor oil or magnesium sulfate), may be employed to diminish uptake or facilitate radioisotope clearance.

Oral intake of potassium iodide can protect individuals from the harmful effects of radioiodine by competitively inhibiting its uptake in the thyroid gland. Proper dosing is essential and varies between adults and children. Incorrect dosing can lead to ineffective protection or potential health risks [48,49]. For example, a dosage too low may not provide adequate protection, while a dosage too high may cause adverse effects such as thyroid dysfunction, particularly in children [49]. Chelating agents such as diethylenetriaminepentaacetate (DTPA) in zinc or calcium salts assist in the removal of radioisotopes of rare earths and actinides such as californium, plutonium, and americium. Sodium bicarbonate is used to manage renal chemical toxicity caused by uranium, which is typically more hazardous than its radiologic toxicity [49]. Prussian blue, which is insoluble ferric III hexacyanoferrate II, is the preferred treatment for cesium-137 and isotopes of thallium [49]. Decisions to treat internal contamination depend on factors such as the dose, and the age.

Management of Acute Radiation Syndrome

Medical management of the ARS certainly requires a multidisciplinary clinical approach, involving a team of healthcare professionals from different specialties such as oncology, hematology, infectious disease, gastroenterology, and others to provide comprehensive and coordinated care. This collaborative approach ensures that all aspects of the patient's health are addressed effectively. The management of ARS is determined by the level and impact of radiation exposure. Treatment options include prophylactic, therapeutic, and palliative care, and can be classified into three categories: supportive treatment, substitution therapy, and aggressive supportive treatment. Supportive treatment involves minimal interventions such as hydration, antiemetic therapy, pain relief, and prophylactic antibiotics. Substitution therapy involves red cell and platelet substitution [50,51]. The use of the thrombomimetics or cytokines; such as granulocyte colony-stimulating factor (G-CSF), is considered standard for ARS patients, to reduce neutropenia and thrombocytopenia [52]. However, the use of stem cell transplantation is an expert decision and is very limited, only for patients who have no other organ systems that will not recover [51,52]. The primary objective of treatment is to support the autologous or stimulated recovery of the suppressed bone marrow and prevent or manage infectious/hemorrhagic complications of ARS [53]. In cases where the severity of radiation exposure has led to irreversible and life-limiting health consequences, some patients may qualify for palliative care, focusing on symptom management and improving their overall quality of life [50,51].

**Figure 3:**
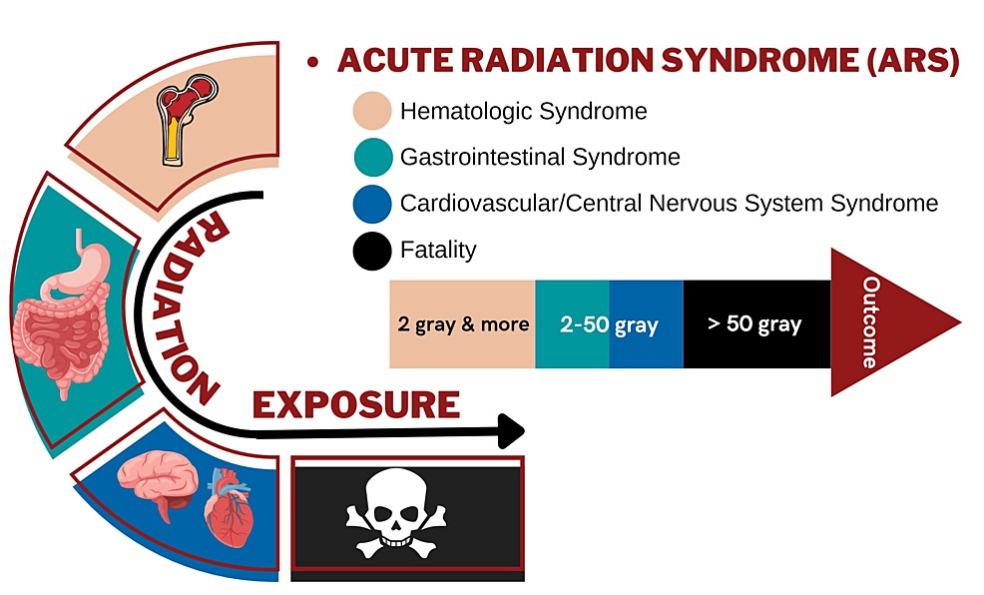
Syndromic Presentation for Individuals with Acute Radiation Syndrome This figure is the original work of the authors.

The management of the impacted system involves focused interventions for particular organs (Figure 3).

For instance, addressing the gastrointestinal tract necessitates administering hydration, antiemetics, antidiarrheals, and selecting appropriate antibiotics due to the fatal consequences of sepsis and intestinal bacteria [54]. Meanwhile, supportive measures are advised for the cerebrovascular system, including antiemetic therapy, anti-seizure drugs, analgesics, and corticosteroids [31].

Management of Cutaneous Radiation Injury

Cutaneous radiation injury (CRI) is a unique condition that can occur not only due to radiation exposure incidents but also during radiation therapy or advanced diagnostic radiology techniques (like computed tomography). The aim of managing CRI is to minimize the severity of the injury and improve outcome [31]. The severity of the injury depends on the level of radiation exposure and can range from mild erythema to severe skin ulceration and scarring (Table 3).

**Table 3:** Phases of Cutaneous Radiation Injuries Adapted from reference [28].

Phase Name	Details	Days Elapsed Since Initial Exposure
Erythema	Reddening of the skin due to inflammation, often appearing as a sunburn-like reaction.	0-2 days
Latent phase	A symptom-free interval where no visible injury is evident.	2-20 days
Acute dermal radionecrosis	Severe skin damage including necrosis, ulceration, and possible infection.	21-60 days
Outcome: Chronic dermal radionecrosis	Persistent skin damage, fibrosis, and potential for secondary cancers.	>60 days
Outcome: Healing	Recovery of the skin with possible residual effects like scarring or pigmentation changes.	>60 days

Early diagnosis is crucial in providing prompt medical attention. The identification of CRI requires a direct observation of the skin, with signs like erythema, blistering, or ulceration being indicative of the condition [31]. Firstly, symptomatic treatment is essential for providing immediate relief. This includes addressing symptoms as they manifest, such as wound care to manage open wounds, pain management to alleviate discomfort, and infection control to prevent and combat infections. Also, topical ointments are a valuable option. These ointments typically contain corticosteroids, locally acting antibiotics, and vitamins. Their application aids in reducing inflammation, promoting the healing of damaged skin, and guarding against infections. Hyperbaric oxygen therapy (HBOT) is another viable approach [31,55]. In HBOT, patients breathe pure oxygen in a pressurized chamber. This therapy enhances blood flow to the affected areas, reduces inflammation, and accelerates the healing process, making it a valuable tool in the management of radiation injuries [55].

In addition to these individual strategies, multidisciplinary therapies have been explored, both in laboratory and clinical trial settings. These therapies involve a combination of treatments, including surgery to remove damaged tissue, radiation therapy to target residual radiation, and chemotherapy to address systemic effects. Such comprehensive approaches are being investigated for their potential to improve patient outcomes [28].

Outpatient Settings: Long-Term Management

In the aftermath of a radiation emergency, high-quality psychological support and long-term management are crucial for both patients who survived the accident and witnesses who may have been exposed to radiation but did not require hospital admission [56]. Patients who have been affected by radiation require long-term psychological assistance to cope with the traumatic effects of radiation exposure. Pregnant patients and parents need specific psychoeducation and support to reduce their anxiety and fear about the effects of radiation on their health and their children's health [57]. Witnesses who have not been hospitalized also need to be provided with psychological support and reassurance to prevent the onset of psychological disorders, including post-traumatic stress disorder [58]. It is also important to take into account the cultural, ethnic, or religious backgrounds of individuals in communicating potential health risks and ensuring that adequate support groups are available. Effective management of rumors and raising public awareness are crucial in mitigating public panic and anxiety [57]. The provision of psychological first aid to affected individuals should involve addressing information needs, safety concerns, physiological needs, emotional support, and prevention of negative social reactions. Furthermore, intensified surveillance after radiation exposure is essential. This involves ongoing monitoring of affected individuals for potential late effects of radiation exposure and providing continuous support to address any emerging physical or psychological issues over time [56-58].

Crisis management in radiation emergencies

Radiation Protection Governing Bodies

Emergency response and radiation preparedness are the joint responsibilities of national governing bodies and regulatory authorities, international governing bodies, and local societies. At the national level, governing bodies and regulatory authorities are primarily responsible for overseeing and coordinating emergency response efforts. International organizations like the International Atomic Energy Agency (IAEA) and the World Health Organization (WHO) offer guidance, technical expertise, and training to enhance national capabilities in managing radiation emergencies [59].

Large-scale incidents often require the involvement of national or international emergency governing bodies [60]. Conversely, local hospitals, regulatory authorities, and emergency services are sufficient to manage minor incidents [60]. Both the IAEA and WHO, along with regional counterparts, aid in knowledge dissemination and awareness-raising about radiation safety and emergency preparedness through various programs such as conferences, workshops, and capacity-building initiatives [59]. The implementation of these activities enhances technical capabilities and instills a culture of preparedness, as well as adequate equipment to handle radiation emergencies.

Integrated Management System (IMS) for Disasters

The implementation of an IMS for radiation safety varies across countries [61,62]. Some nations have established self-sufficient IMS, primarily due to their development or launch of peaceful nuclear reactors. These nations have demonstrated their commitment to radiation safety by creating comprehensive management systems that cover various areas, including radiation protection, emergency response, and safe radiation source usage. The integration of these components aims to establish effective and coordinated measures for preventing and responding to radiation-related incidents or disasters.

Best Practices for Optimal Radiation Response

Building an effective radiation defense system is a challenging undertaking that demands significant national and international cooperation. While some countries are making significant progress in constructing their nuclear infrastructure, others face financial and political challenges [63], which can hinder radiation safety planning. The following recommendations aim to enhance the overall quality of the radiation defense system on an international level.

National Stockpiles for Radiation Emergencies

Availability of the medical countermeasures and antidots is critical in managing radiation injuries. The WHO strongly advises its member states to establish national stockpiles or at least have access to neighboring stockpiles [64]. Establishing a national stockpile should be based on the most likely scenarios. When setting up a stockpile, it is important to consider various approaches, such as the size of the population that may be impacted by different scenarios and the available resources and capabilities of the health system in the country [64]. As storage and maintenance are necessary, products that have minimal refrigeration requirements and long shelf lives are preferred [64]. Clinical evidence about the use of blocking and decorporating agents, as well as cytokines, is limited [64]. Based on experience from past accidents, only a few agents have proven to be effective for treating radiation injuries and internal radionuclide contamination [64,65]. These agents approved in several countries as medical countermeasures for radionuclide incorporation or radiation injury-related indications included potassium iodine, Prussian blue (ferric ferrocyanide), Ca, or Zn DTPA [64]. During a nuclear accident, inhalation of contaminated air and ingestion of contaminated food and drinking water may lead to internal exposure and uptake of radioactive iodine mainly by the thyroid gland. Oral administration of stable iodine is referred to as iodine thyroid blocking and is considered an appropriate strategy for reducing the risk of thyroid cancer. Oral Prussian blue capsules were approved for the treatment of internal contamination with radioactive cesium. Ca and Zn DTPA administered intravenously or by nebulizer is used to treat contamination with transuranic radionuclides (e.g., Pu, Am, and Cm) [64,65].

Maintenance of a stockpile is another issue that should be considered. It requires frequent monitoring and evaluation, and the formulary must be regularly reviewed and updated. Quality assurance and quality control measures must be applied continually to maintain the currency, accuracy, and completeness of the stockpile. A protocol for a stockpile and decision-making should include criteria for triage and setting priorities for allocation and distribution in cases of limited availability of medical countermeasures [64].

A clear communications strategy is also necessary for the management of a stockpile, such as for an explanation of timelines and priorities for access to certain products.

Medical and Paramedical Personnel

Medical and paramedical personnel refer to professionally trained healthcare workers, including doctors, nurses, dentists, physiotherapists, and other allied health professionals, who provide medical care and treatment to patients. During the management of a radiation emergency, medical and paramedical personnel play a crucial role in ensuring the prompt and effective treatment of individuals affected by radiation exposure or contamination.

Education and Training

Education and training play critical roles in effective radiation protection. Healthcare providers and first responders must receive comprehensive training to effectively diagnose and triage cases based on the type, origin, severity, and urgency of radiation exposure [33]. Knowledge of various types of radiation and their effects on the body is also mandatory. In parallel, adequate training in managing patients with different levels of radiation exposure and utilizing safety equipment is essential.

Application of Safety Principle

**Figure 4:**
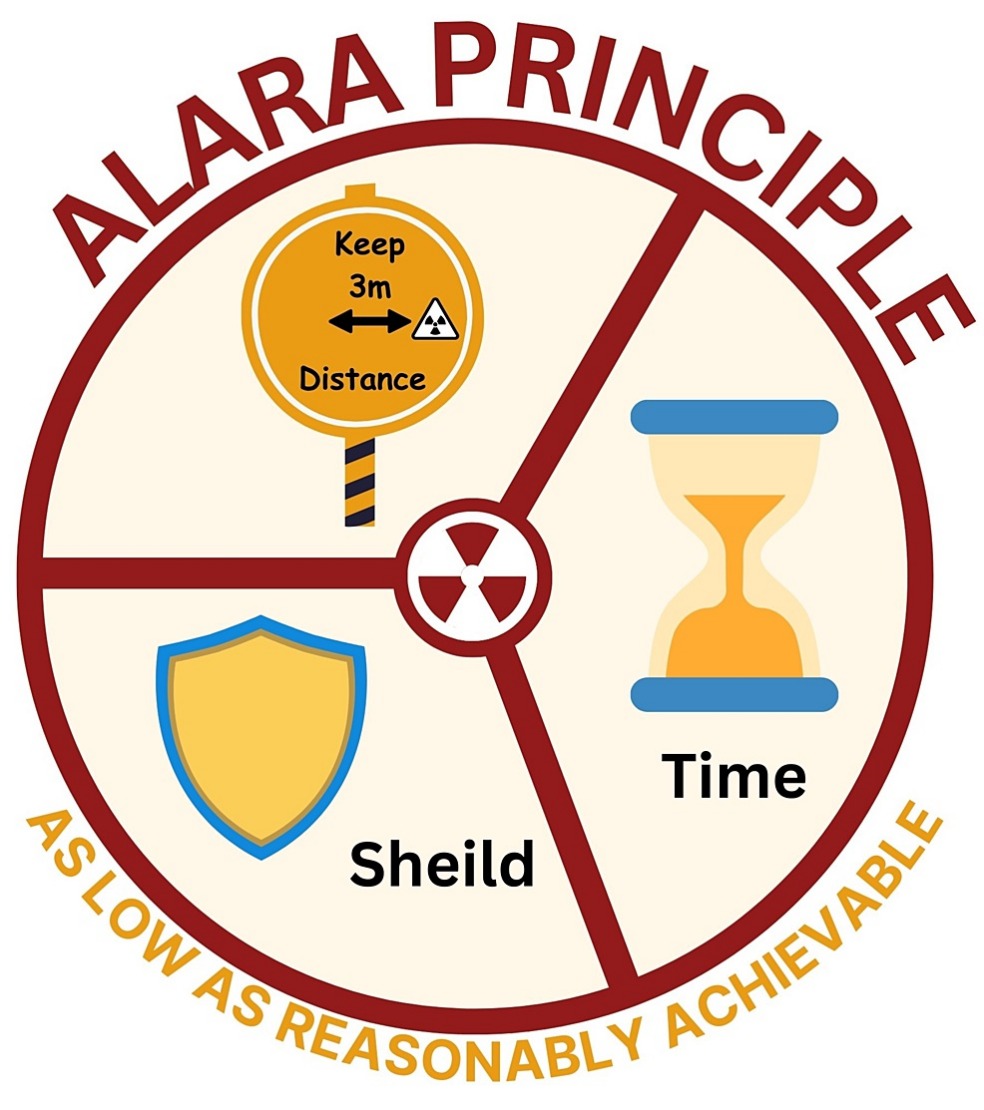
Demonstration of the Principle of As Low As Reasonably Achievable (ALARA) This figure is the original work of the authors.

Safety principles must be applied to ensure a safe working environment. These principles include the use of PPE, monitoring devices, and efficient decontamination procedures. Radiation protection is the principle of As Low As Reasonably Achievable (ALARA) [66]. Methods to reduce radiation exposure include limiting duration, minimizing exposure time, increasing distance, and implementing shielding. Additionally, personnel should understand the difference between radiation exposure and contamination. Adherence to the ALARA principles can significantly decrease the likelihood of contamination (Figure 4).

Upgrading Medical Equipment and Facilities

The development of a comprehensive plan that considers the infrastructure, technology, and resources available in each country is essential. Hospitals and medical facilities must have the necessary equipment and supplies to diagnose and treat patients exposed to radiation, such as PPE, decontamination equipment, monitoring equipment, and personal alarming dosimeters. Whole body counter should be used to estimate radionuclide deposition in various tissues/organs and to predict the risk of internal radiation before triage of residents. A high-speed screening system for radioactivity in foods was developed to measure the concentration of radioactive cesium (Bq/kg). The system is useful to avoid internal radiation due to contaminated foods [67].

Upgrading the response system usually starts by allocating financial resources to improve radiation emergency infrastructure, and investing in cutting-edge medical technologies and equipment.

## Conclusions

In brief, radiation emergencies pose serious risks. Effective response requires a well-structured plan and collaboration among first responders. This review comprehensively addresses the multifaceted challenges and critical strategies essential for effective radiation emergency preparedness and response. It underscores the importance of a well-coordinated approach involving education, infrastructure, and rapid response mechanisms to mitigate the impacts of radiation exposure. The review also highlights the role of bioindicators in assessing exposure levels and the necessity for advanced medical and protective resources to safeguard public health and safety. Through detailed analysis and proposed recommendations, this study contributes significantly to enhancing the readiness and response to radiation emergencies, ultimately aiming to protect both individuals and communities from the potentially devastating effects of ionizing radiation.

## References

[1] Yamamoto LG (2013). Risks and management of radiation exposure. Pediatr Emerg Care.

[2] Li C, Ansari A, Etherington G (2016). Managing internal radiation contamination following an emergency: Identification of gaps and priorities. Radiat Prot Dosimetry.

[3] Rump A, Becker B, Eder S, Lamkowski A, Abend M, Port M (2018). Medical management of victims contaminated with radionuclides after a "dirty bomb" attack. Mil Med Res.

[4] Wodarz D, Sorace R, Komarova NL (2014). Dynamics of cellular responses to radiation. PLoS Comput Biol.

[5] Port M, Majewski M, Abend M (2019). Radiation dose is of limited clinical usefulness in persons with acute radiation syndrome. Radiat Prot Dosimetry.

[6] Guan B, Li D, Meng A (2023). Development of radiation countermeasure agents for acute radiation syndromes. Animal Model Exp Med.

[7] Fliedner TM (2008). Medical Management of Radiation Accidents. Manual on the Acute Radiation Syndrome.

[8] Bauchner H, Fontanarosa PB, Livingston EH (2020). Conserving supply of personal protective equipment-A call for ideas. JAMA.

[9] Paretzke HG (1988). The impact of the Chernobyl accident on radiation protection. Health Phys.

[10] Karmaker N, Maraz KM, Islam F (2021). Fundamental characteristics and application of radiation. GSC Adv Res Rev.

[11] Donya M, Radford M, ElGuindy A, Firmin D, Yacoub MH (2014). Radiation in medicine: Origins, risks and aspirations. Glob Cardiol Sci Pract.

[12] Hirama T, Tanosaki S, Kandatsu S (2003). Initial medical management of patients severely irradiated in the Tokai-mura criticality accident. Br J Radiol.

[13] Beylergil V, Carrasquillo JA, Weber W, Larson SM (2013). A Historical Perspective on the Use of Radionuclides for Therapy. Therapeutic Nuclear Medicine.

[14] Hirano S, Komatsu M, Miura S (2022). Radioactive materials released by the Fukushima nuclear accident. Forest Radioecology in Fukushima.

[15] Yasunari TJ, Stohl A, Hayano RS, Burkhart JF, Eckhardt S, Yasunari T (2011). Cesium-137 deposition and contamination of Japanese soils due to the Fukushima nuclear accident. Proc Natl Acad Sci U S A.

[16] Mück K, Sinojmeri M, Whilidal H, Steger F (2001). The long-term decrease of 90Sr availability in the environment and its transfer to man after a nuclear fallout. Radiat Prot Dosimetry.

[17] Reiners C, Drozd V, Yamashita S (2020). Hypothyroidism after radiation exposure: Brief narrative review. J Neural Transm (Vienna).

[18] Al‐Ibraheem A, Abdlkadir AS, Albalooshi B (2022). Theranostics in the Arab World; Achievements & Challenges. Jordan Med J.

[19] Obrador E, Salvador-Palmer R, Villaescusa JI, Gallego E, Pellicer B, Estrela JM, Montoro A (2022). Nuclear and radiological emergencies: Biological effects, countermeasures and biodosimetry. Antioxidants (Basel).

[20] Boice JD Jr, Lubin JH (1997). Occupational and environmental radiation and cancer. Cancer Causes Control.

[21] Lowe D, Roy L, Tabocchini MA, Rühm W, Wakeford R, Woloschak GE, Laurier D (2022). Radiation dose rate effects: What is new and what is needed?. Radiat Environ Biophys.

[22] Hamada N, Azizova TV, Little MP (2020). An update on effects of ionizing radiation exposure on the eye. Br J Radiol.

[23] Mainprize JG, Yaffe MJ, Chawla T, Glanc P (2023). Effects of ionizing radiation exposure during pregnancy. Abdom Radiol.

[24] Yanovskiy M, Shaki YY, Socol Y (2019). Ethics of adoption and use of the linear No-Threshold model. Dose Response.

[25] Jacob S, Michel M, Spaulding C (2010). Occupational cataracts and lens opacities in interventional cardiology (O'CLOC study): Are X-Rays involved? Radiation-induced cataracts and lens opacities. BMC Public Health.

[26] Stenke L, Hedman C, Lagergren Lindberg M, Lindberg K, Valentin J (2022). The acute radiation syndrome-need for updated medical guidelines. J Radiol Prot.

[27] López M, Martín M (2011). Medical management of the acute radiation syndrome. Rep Pract Oncol Radiother.

[28] Iddins CJ, DiCarlo AL, Ervin MD, Herrera-Reyes E, Goans RE (2022). Cutaneous and local radiation injuries. J Radiol Prot.

[29] Miller RW (1995). Delayed effects of external radiation exposure: A brief history. Radiat Res.

[30] Xu Y, Wang C, Chen S, Lu B, Zhou J (2023). Nuclear Blast Injury. Explosive Blast Injuries.

[31] Mettler FA Jr, Voelz GL (2002). Major radiation exposure—what to expect and how to respond. N Engl J Med.

[32] Arora R, Chawla R, Marwah R (2010). Medical radiation countermeasures for nuclear and radiological emergencies: Current status and future perspectives. J Pharm Bioallied Sci.

[33] Lim H, Ng KS, Tan HH, Leong KWG (2011). Hospital preparedness for radiation emergencies and medical management of multiple combined radiation injury victims. Proc Singap Healthc.

[34] Wolbarst AB, Wiley AL Jr, Nemhauser JB, Christensen DM, Hendee WR (2010). Medical response to a major radiologic emergency: A primer for medical and public health practitioners. Radiology.

[35] (2024). WHO guidance on preparing for national response to health emergencies and disasters. https://www.who.int/publications/i/item/9789240037182.

[36] International Health Regulations (2005) (2024). WHO: International Health Regulations (2005) - Third edition. World Health Organization.

[37] International Atomic Energy Agency (2024). Criteria for use in preparedness and response for a nuclear or radiological emergency. Criteria for use in preparedness and response for a nuclear or radiological emergency.

[38] Ramesh AC, Kumar S (2010). Triage, monitoring, and treatment of mass casualty events involving chemical, biological, radiological, or nuclear agents. J Pharm Bioallied Sci.

[39] Sasser S (2006). Field triage in disasters. Prehosp Emerg Care.

[40] Blakely WF, Port M, Abend M (2021). Early-response multiple-parameter biodosimetry and dosimetry: risk predictions. J Radiol Prot.

[41] Rojas-Palma C: Ionizing RadiationTMT Handbook (2024). WHO: TMT handbook : Triage, monitoring and treatment of people exposed to ionizing radiation following a malevolent act. https://www.who.int/publications/m/item/tmt-handbook.

[42] Fliedner TM, Dörr H, Meineke V (2005). Multi-organ involvement as a pathogenetic principle of the radiation syndromes: A study involving 110 case histories documented in SEARCH and classified as the bases of haematopoietic indicators of effect. Br J Radiol.

[43] Dainiak N (2018). Medical management of acute radiation syndrome and associated infections in a high-casualty incident. J Radiat Res.

[44] Ohba T, Tanigawa K, Liutsko L (2021). Evacuation after a nuclear accident: Critical reviews of past nuclear accidents and proposal for future planning. Environ Int.

[45] Bushberg JT, Kroger LA, Hartman MB, Leidholdt EM Jr, Miller KL, Derlet R, Wraa C (2007). Nuclear/radiological terrorism: Emergency department management of radiation casualties. J Emerg Med.

[46] Anderson RM (2019). Cytogenetic biomarkers of radiation exposure. Clin Oncol.

[47] Schleipman AR, Gerbaudo VH, Castronovo FP (2004). Radiation Disaster Response: Preparation and simulation experience at an academic medical center. J Nucl Med Technol.

[48] Ilias I, Rizzo M, Meristoudis G (2023). Potassium Iodide in nuclear accidents: Give it timely, swiftly and judiciously. Endocr Metab Immune Disord Drug Targets.

[49] Domínguez-Gadea L, Cerezo L (2011). Decontamination of radioisotopes. Rep Pract Oncol Radiother.

[50] Zuziak P, Bielaska A (2023). Acute radiation syndrome. J Educ, Health Sport.

[51] Nair V, Karan DN, Makhani CS (2017). Guidelines for medical management of nuclear/radiation emergencies. Med J Armed Forces India.

[52] MacVittie TJ, Farese AM, Jackson W 3rd (2005). Defining the full therapeutic potential of recombinant growth factors in the post radiation-accident environment: The effect of supportive care plus administration of G-CSF. Health Phys.

[53] Horta ZP, Case CM Jr, DiCarlo AL (2019). Use of growth factors and cytokines to treat injuries resulting from a radiation public health emergency. Radiat Res.

[54] Dainiak N, Gent RN, Carr Z (2011). Literature review and global consensus on management of acute radiation syndrome affecting nonhematopoietic organ systems. Disaster Med Public Health Prep.

[55] Creutzberg CL (2015). Hyperbaric oxygen therapy and radiation-induced injuries. Lancet Oncol.

[56] Berger ME, Christensen DM, Lowry PC, Jones OW, Wiley AL (2006). Medical management of radiation injuries: Current approaches. Occup Med.

[57] Gale RP, Armitage JO, Hashmi SK (2021). Emergency response to radiological and nuclear accidents and incidents. Br J Haematol.

[58] Nor A, Rozilawati A, Farah N, Khadijah K, Farihan J, Ahmad S (2022). The knowledge and perception of radiation therapists on psychosocial and supportive care for cancer patients in Hospital Canselor Tuanku Mukhriz and National Cancer Institution Malaysia. Journal of Medical Imaging and Radiation Sciences.

[59] Vassileva J, Applegate K, Paulo G, Vano E, Holmberg O (2022). Strengthening radiation protection education and training of health professionals: conclusions from an IAEA meeting. J Radiol Prot.

[60] Majali MM (2024). Majali MM: SAFETY OF RADIATION SOURCES AND OTHER RADIOACTIVE MATERIALS IN JORDAN. Safety of radiation sources and other radioactive materials in Jordan. (2001). Accessed.

[61] Berris T, Žontar D, Rehani MM (2017). Survey on impact of regulations on radiation safety and development of radiation safety culture in 25 countries. J Med Imaging.

[62] Gibaud B, Brenet M, Pasquier G (2021). A semantic database for integrated management of image and dosimetric data in low radiation dose research in medical imaging. AMIA Annu Symp Proc.

[63] Al-Ibraheem A, Abdlkadir AS, Mohamedkhair A, Mikhail-Lette M, Al-Qudah M, Paez D, Mansour AH (2022). Cancer diagnosis in areas of conflict. Front Oncol.

[64] (2024). WHO: National stockpiles for radiological and nuclear emergencies: Policy advice. National stockpiles for radiological and nuclear emergencies: policy advice.

[65] Zhang Y, Sadgrove MP, Mumper RJ, Jay M (2013). Radionuclide decorporation: Matching the biokinetics of actinides by transdermal delivery of pro-chelators. AAPS J.

[66] Musolino SV, DeFranco J, Schlueck R (2008). The ALARA principle in the context of a radiological or nuclear emergency. Health Phys.

[67] Mizuta T, Tachibana K, Kobayashi S (2012). Development of FOODSEYE, a high-speed screening system for radioactivity in foods. Shimadzu Hyoron.

